# Next-Generation Sequencing Reveals a Diagnostic and Prognostic Role of the *TP53* R273C Mutation in Lower-Grade, *IDH*-Mutant Astrocytomas

**DOI:** 10.3390/ijms262311483

**Published:** 2025-11-27

**Authors:** Lara Navarro, Javier Megías, Irene Salazar-Saura, Moisés Sánchez-Pardo, Esther Roselló-Sastre, Teresa San-Miguel

**Affiliations:** 1 Pathology Service, Consorcio Hospital General Universitario de Valencia, 46014 Valencia, Spain; lara.navarro@uv.es (L.N.); salazar_ire@gva.es (I.S.-S.); rosello_est@gva.es (E.R.-S.); 2 Research Group on Tumors of the Central Nervous System, Pathology Department, University of Valencia, 46010 Valencia, Spain; 3 INCLIVA Foundation, 46010 Valencia, Spain; 4 Department of Neurosurgery, Consorcio Hospital General Universitario de Valencia, 46014 Valencia, Spain; moises.s.pardo@gmail.com

**Keywords:** lower-grade, IDH-mutant astrocytomas, *TP53* R273C mutation, next-generation sequencing, Ki-67, TCGA

## Abstract

The current WHO grading of central nervous system tumors relies exclusively on histopathological criteria for diagnosing lower-grade, IDH-mutant astrocytomas (LGIMAs), overlooking genetic features. The *TP53* R273C mutation, frequently observed in brain tumors, may influence LGIMA biology and aggressiveness. We analyzed 14 *TP53*-mutant LGIMAs using NGS. Five tumors (33.3%) carried the R273C mutation; these were mostly of grade 2 and all from female patients. Ki-67 levels in R273C-mutant tumors were higher compared with those in other *TP53*-mutant grade 2 tumors but lower than those in grade 3 tumors, which may suggest that R273C defines a more aggressive grade 2 profile. This mutation was linked to loss of the wild-type allele, supporting a loss-of-function mechanism. Its frequency was found to be potentially higher in women, and this sex-based difference reached statistical significance when incorporating TCGA LGIMA data. Overall, the R273C mutation, although mechanistically unclear, is more prevalent than other *TP53* variants and defines a distinct biological subset of LGIMAs, marked by increased Ki-67 and female predominance. Incorporating *TP53* and broader genetic profiling via NGS could improve our understanding of LGIMAs and support a refined classification system, enhancing diagnostic and prognostic accuracy.

## 1. Introduction

Infiltrative astrocytic tumors are the most frequent primary malignant neoplasms of the central nervous system (CNS), and the lack of effective therapeutic approaches against them explains their high mortality and morbidity rates [1]. Astrocytomas show a wide range of biologic behavior and different outcomes among the four grades in which they are classified by the World Health Organization (WHO) system, from pilocytic astrocytoma (grade 1) to glioblastoma (grade 4) [2]. In the latest updates, this classification tends to highlight genetic tumor patterns as critical features in order to determine the grades of these entities. Thus, mutations in the isocitrate dehydrogenase genes *IDH1* and *IDH2* define specific entities, associated with lower histologic grades and better prognosis when compared to *IDH*-wild-type astrocytomas [3].

*IDH*-mutant astrocytomas (IMAs) are diffusely infiltrating gliomas that frequently present mutations in other genes, *TP53* (in over 90% of tumors) and *ATRX* (in over 70% of tumors), and do not show the 1p/19q codeletion [4,5].

The ubiquity of *TP53* mutations suggests that they play an essential role in the initiation and progress of growth in these relatively less aggressive tumors, which is an interesting and unexplained issue, considering that other brain and non-CNS tumors show highly malignant behavior when *TP53* mutations are present. For instance, fewer than 30% of *IDH*-wild-type glioblastomas show *TP53* mutations; however, their acquisition occurs later in tumor progression, their behavior is more aggressive, and their grade is higher [2,6,7], thus suggesting a different and critical role of *TP53* mutations in IMA. Of all the mutations described for this locus, there is a single dominant hotspot at codon 273, which accounts for 38% of all *TP53* mutations in IMA, whereas its frequency in *IDH*-wild-type tumors is much lower, around 5% [3]. Moreover, of all the codon 273 *TP53* mutations, there is one that stands out for its high prevalence: the one that codes for the R273C amino acid substitution or *TP53* R273C. This mutation accounts for 20 to 30% of all *TP53* mutations in IMA [3].

According to the WHO grading system for CNS tumors, IMA can be classified into three grades: 2, 3 and 4. Grades 2 and 3 comprise low-grade, *IDH*-mutant astrocytomas (LGIMAs), which have less aggressive behavior than grade 4 gliomas [2]. Considering prognosis, some LGIMAs are stable for a long time, while others progress to higher degrees fast, within months [8].

Grade 4 *IDH*-mutant astrocytomas are discriminated from their LGIMA counterparts based on the presence of the homozygous deletion of *CDKN2A*/*2B*, the existence of microvascular proliferation or necrosis, or any combination of these three features. Instead, LGIMAs are classified according to the absence of any of these grade 4 features and histopathologic criteria.

**Figure 1:**
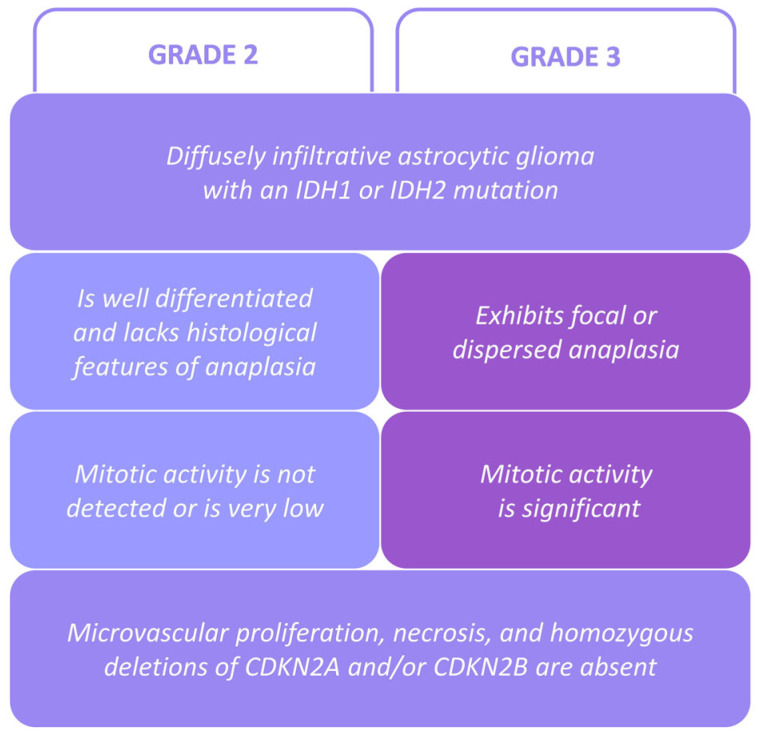
LGIMA classification criteria according to the 2021 WHO grading system [2].

Grade 2 *IDH*-mutant astrocytoma is an LGIMA with a lower grade and thus a less aggressive one. The histopathologic features that define it are (i) good differentiation (low cell density, no anaplastic features) and (ii) absence or a very low rate of mitotic and proliferative activity (Figure 1) [2]. However, the histopathological classification of grades 2 and 3 cannot accurately predict aggressiveness nor survival. Additional genetic characterization and classification could be useful in order to better estimate aggressiveness and guide treatment [9]. *TP53* is one of the most frequently mutated genes in LGIMAs, with the *TP53* R273C mutation being predominant [10,11]. The biological role of *TP53* R273C is still not well understood. In several cancers and in vitro models with tumor cells, this mutation has shown contradictory effects [12,13,14,15]. In LGIMAs, it seems clear that *TP53* R273C may play a fundamental role in aggressiveness, and it has been postulated that this mutation could be an unfavorable prognostic biomarker for LGIMA patients and associated with higher tumor mutation burden values [9].

The *ATRX* mutation, the second main mutation present in IMA, is associated with an abnormal telomere maintenance mechanism known as alternative lengthening of telomeres. It is known that this mutation can induce p53-dependent cell death in some contexts. Therefore, *TP53* mutations in IMA may enable tumor cell survival in the setting of *ATRX* loss [16].

In the present study, we describe a cohort of 14 patients with LGIMAs harboring *TP53* mutations, with a long clinical follow-up, and we assess whether these mutations had a beneficial or malignant effect on the prognosis or our patients, considering all other gene mutations which could be simultaneously present.

The predominant mutation in our cohort, R273C, appears to define a more aggressive tumor subtype, with higher prevalence in female patients, compared to other LGIMAs lacking this mutation. Similarly to R273C, reported mutations in *TP53* or in other genes may also play a role in determining LGIMA phenotype, yet they are not considered under the current WHO classification criteria. Therefore, we propose next-generation sequencing (NGS) as a valuable tool for improving characterization and classification of LGIMAs, with the potential to better predict tumor behavior and patient survival.

## 2. Results

From our neuropathology archives, we retrieved data on fourteen patients diagnosed with LGIMAs, which were classified as grade 2 or 3, according to the WHO 5th Edition grading system criteria (Figure 1) [2]. Our cohort included five men and nine women (two cases were from the same woman, with a surgical resected relapsed tumor), with ages from 29 to 70 years old (45.6 ± 12.8), without significant differences between sexes (45.0 ± 14.9 in men against 46.0 ± 12.5 in women). The median follow-up was 88 months (with a range of 24–240 months). All patients were alive at the end of this study.

**Figure 2:**
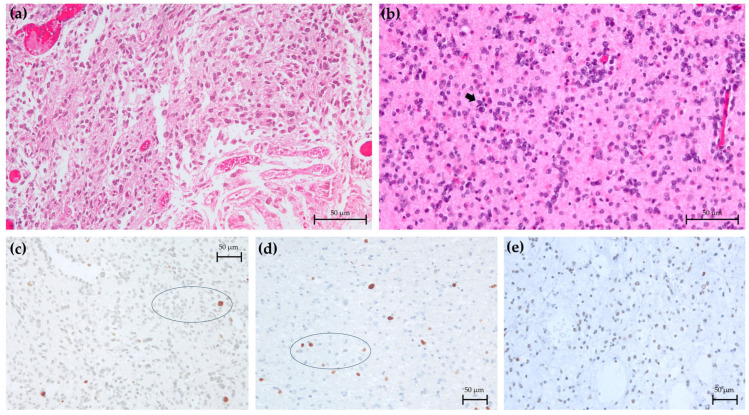
*IDH*-mutant astrocytomas of grades 2 and 3, demonstrating histological differences: (**a**) Absence of anaplasia and mitotic figures in grade 2 (HE). (**b**) Focal anaplasia and low mitotic activity (arrow) in grade 3 (HE). (**c**) Low Ki67 in grade 2 (2%). Oval indicates representative area where Ki-67 expression is detected. (**d**) Higher Ki67 levels in grade 3 (12%). Oval indicates representative area where Ki-67 expression is detected. (**e**) Absence of ATRX expression in both types of LGIMA. Micrographs are representative of samples of our cohort. Magnification: 40×. HE: hematoxylin–eosin.

Ten LGIMAs from our cohort were diagnosed as grade 2 *IDH*-mutant astrocytomas, while the other four were diagnosed as grade 3. Histologically, they were diffuse glial cell tumors with low/moderate density, mild or medium nuclear atypia, and infiltrative growth. No mitotic figures were detected in grade 2 tumors, whereas there were some mitosis or anaplastic features in grade 3 tumors (Figure 2a,b). Neither necrosis nor vascular proliferation was detected.

Immunohistochemistry showed low Ki-67 values in grade 2 tumors (range of 1–7%, average of 4%), whereas grade 3 tumors had higher levels (range of 5–12%, average of 9.0%). Nuclear p53 expression was present in all cases (range of 1–80%, average of 45%). We observed ATRX in nine cases: seven cases lost nuclear expression of ATRX, but two cases were focally positive. These two cases and the five ones with no available immunohistochemical information were studied for 1p/19q codeletion, confirming a negative result (no co-deletion present) (Figure 2c–e).

**Figure 3:**
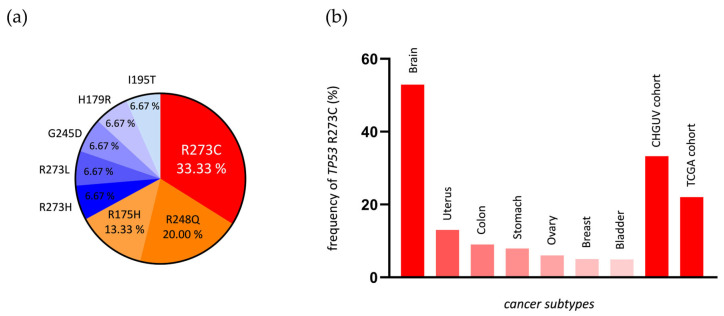
(**a**) Frequency of *TP53* mutations in the LGIMA tumors of our cohort. (**b**) Predominance of the *TP53* R273C mutation in brain tumors compared to cancers from other tissues, determined through TCGA studies.

Table 1 summarizes the most relevant features of the 14 cases of our cohort, including *IDH1/2* and *TP53* mutations. Molecular diagnosis via NGS confirmed *IDH1* R132H (in 11 tumors), *IDH1* R132G (1 tumor), *IDH1* R132L (1 tumor), and *IDH2* R172G (1 tumor) mutations. All the 14 LGIMAs presented *TP53* mutations, most of them at the hotspot codon 273 (46.6% of the mutations), with a median variant allele frequency (VAF) of 59,5% (13–96%). Five tumors presented the *TP53* R273C mutation (33.3%), three the *TP53* R248Q mutation (20%), and two the *TP53* R175H mutation (13.3%), and the other four showed different *TP53* mutations (Table 1, Figure 3a). Association with Li–Fraumeni syndrome was ruled out for all the samples.

We performed a TCGA analysis of only LGIMA *IDH1/2* and *TP53*-mutant tumors. Of 115 LGIMAs with both mutations (TCGA cohort), 25 tumors presented the R273C mutation (21.7%). The percentage of tumors with this mutation in our cohort (33.3%) is in line with that in the TCGA cohort (21.7%), despite being higher; it is also congruent with the frequency in all brain tumors, in which the hotspot mutation stands out when compared with that in other cancer types, according to the TCGA database (Figure 3b).

Then, as shown in Table 2, we compared the features of cases with the *TP53* R273C mutation from our cohort with those of other cases, in order to find differences that could be related to this specific mutation in LGIMA. Interestingly, all the five tumors with the *TP53* R273C mutation came from female patients. In contrast, tumors with other *TP53* mutations had a similar distribution between both sexes: five were from men and four from women. Additionally, most of the tumors with the *TP53* R273C mutation were of grade 2 (four of the five), while tumors with other mutations were more evenly distributed between grades 2 (six cases) and 3 (three cases). Tumors with the *TP53* R273C mutation were diagnosed in patients with older ages (51.6 ± 13.9 vs. 42.3 ± 11.7) and showed similar sizes (34.4 cm^3^ ± 27.7 vs. 32.7 cm^3^ ± 47.0) when compared to tumors with other *TP53* mutations.

**Figure 4:**
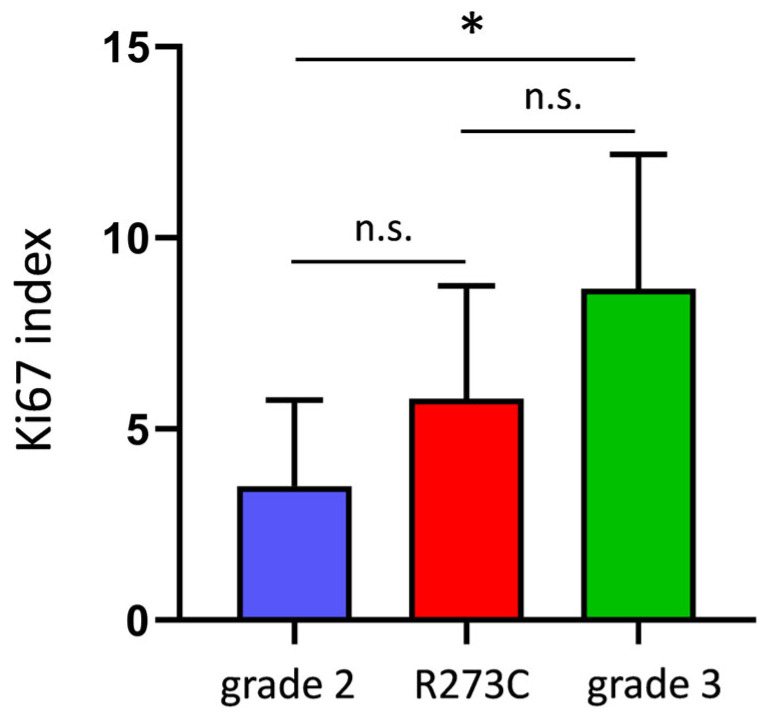
Ki67 levels in grade 2 LGIMA with other mutations in *TP53*, LGIMA with the *TP53* R273C mutation, and grade 3 LGIMA with other mutations in *TP53*. The results are the mean ± S.D. Grade 2: n = 6 cases; R273C: n = 5 cases; grade 3: n = 3 cases. * *p* < 0.05, Student’s *t*-test. n.s.: not significant.

All grade 2 LGIMA tumors had low proliferation indexes, ranging from 1% to 7%, whereas grade 3 LGIMAs had values ranging from 5% to 12%, according to the Ki-67 labeling index. Remarkably, if we consider LGIMAs with the *TP53* R273C mutation as a specific group, independent of the canonical grading, we find that these tumors had Ki67 levels between those of tumors of grades 2 and 3. There was an interesting tendency in cell proliferation between *TP53* R273C LGIMAs and the grade 2 tumors with other *TP53* mutations, since the Ki-67 index was higher in *TP53* R273C tumors than it was in other grade 2 tumors (5.8 ± 3.0 vs. 3.5 ± 2.3), and it was lower in *TP53* R273C tumors than it was in grade 3 LGIMAs (5.8 ± 3.0 vs. 8.7 ± 3.5) (Figure 4). Although these differences are not significant due to the small size of our cohort, the Ki67 values show three levels of cell proliferation if LGIMAs with the R273C mutation are considered a specific category. This finding may suggest the existence of grade 2 LGIMAs with different proliferative behaviors, depending on *TP53* and perhaps other gene mutations, thereby promoting improving the current LGIMA classification through advanced genetic studies via NGS.

**Figure 5:**
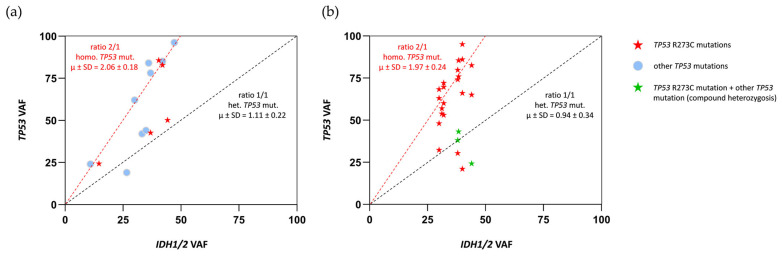
(**a**) VAF ratios between *TP53* and *IDH1/2* mutations for each tumor in our CHGUV cohort. According to these, tumors were identified as homozygous (ratio of 2/1) or heterozygous (ratio of 1/1) for *TP53* mutations. (**b**) VAF ratios between *TP53* R273C and *IDH1/2* mutations for each tumor in the TCGA cohort. According to these, tumors were identified as homozygous (ratio of 2/1) or heterozygous (ratio of 1/1) for *TP53* R273C mutations.

Then, we investigated whether *TP53* R273C mutations were mostly heterozygous or homozygous in our tumors. It is known that *IDH1/2* mutations are ubiquitous in *IDH*-mutant astrocytomas, which means that all tumor cells present a heterozygous mutation of *IDH1* or *IDH2* and retain a wild-type allele, necessary for tumor cell survival. For this reason, the *IDH* mutation frequency in each tumor sample is considered a reference measure of tumor cellularity [17,18]. According to this, we established the ratios between the variant allele frequencies (VAFs) of *TP53* mutations and *IDH1/2* mutations for all tumors, in order to elucidate whether the tumors had homozygous (ratio of 2/1) or heterozygous (ratio of 1/1) *TP53* mutations (Figure 5a). In our cohort, the majority of tumors showed homozygous mutations (9 of 14), which is concordant with data obtained across all kinds of cancers, where it has been shown that the loss of the wild-type *TP53* allele, or second hit, occurs in approximately 90% of cases [19]. *TP53* R273C mutations were homozygous (in three cases of five) and heterozygous (in two of five). Having one or two mutant alleles did not correlate with the Ki-67 index, the size of the tumor, or the main histologic features of the two grades. In order to evaluate the proportions of homozygous and heterozygous R273C mutations in LGIMAs, we studied the VAF ratios between *TP53* R273C and *IDH1/2* mutations in the TCGA cohort. Of the 25 cases with *TP53* R273C, 19 were homozygous (76%), and 6 were heterozygous (24%) (Figure 5b). Remarkably, half of the heterozygous cases (three of six) were compound heterozygotes, with a second deleterious mutation in the other *TP53* allele and, therefore, closer to homozygous features.

**Figure 6:**
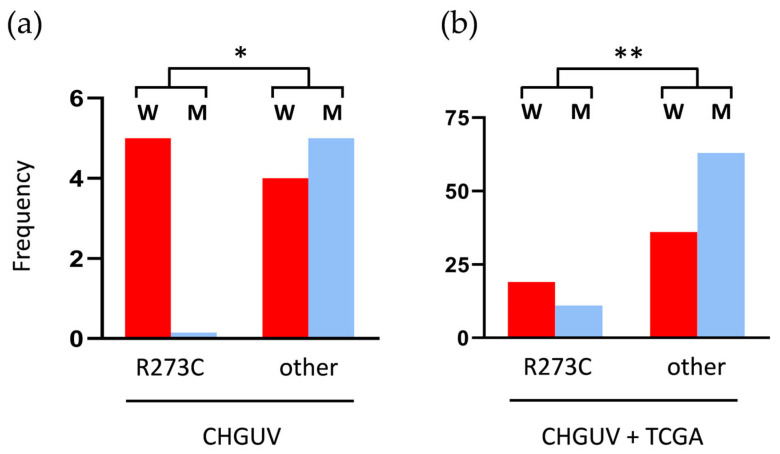
Sex differences related to *TP53* mutations in LGIMAs: (**a**) in all the LGIMAs of our CHGUV cohort; (**b**) in all the LGIMAs of our cohort plus 221 IDH-mutant and *TP53*-mutant LGIMAs from the TCGA database. * *p* < 0.05 and ** *p* < 0.01 (chi-squared test). W: women; M: men.

Previous works revealed that *TP53* R273C mutations are more common in women than in men in *IDH*-mutant astrocytomas of all grades. These mutations account for 26% of all *TP53* mutations in women and only 11% in men [3]. In our cohort, only with LGIMAs, 55.6% of tumors from women had the *TP53* R273C mutation (five cases of nine), and this mutation was not present in any of the tumors from men. In contrast, LGIMAs with other *TP53* mutations were equally distributed between sexes (four in women and five in men). This difference was statistically significant (* *p* < 0.05, chi-squared test) (Figure 6a). In the TCGA LGIMA cohort, 25 tumors of 115 presented the R273C mutation (21.7%), while the other 90 (78.3%) had other *TP53* mutations. The *TP53* R273C mutation was slightly more frequent in women, and the other *TP53* mutations were more frequent in men, but the differences between the two groups were not significant. Interestingly, the addition of our 14 LGIMA cases to the TCGA cohort made the differences significant, as shown in Figure 6b (** *p* < 0.01; chi-squared test). These findings unveil an apparent sex-dependent bias in the presence of *TP53* R273C in *IDH*-mutant astrocytomas and, more prominently, in LGIMAs, which needs to be explored.

## 3. Discussion

The WHO glioma grading system has progressively improved in recent years, increasingly incorporating genetic criteria, which, together with histopathological parameters, allow for a more accurate classification of tumors [2,20]. The stratification of gliomas based on the presence or absence of mutations in *IDH1* or *IDH2* has represented a major advancement in the understanding of glioma biology and has improved diagnostic and prognostic accuracy, to the extent that this criterion alone is sufficient to discriminate between astrocytomas and glioblastomas. Additional genetic alterations widely reported in the literature are now considered in the refined classification of high-grade gliomas, such as mutations in *TP53* and *ATRX* and *EGFR* amplification, further underscoring the predominant role of tumor genetics [21,22,23].

However, regarding LGIMAs, classification still relies exclusively on histopathological criteria and disregards genetic factors. Thus, the principal features distinguishing grade 2 from grade 3 *IDH*-mutant astrocytomas according to the WHO are histological anaplasia and increased mitotic activity. However, no studies on LGIMA cohorts have established a clear mitotic count threshold for stratifying risk between both grades [2]. Similarly, studies assessing proliferation based on the Ki-67 index have not defined criteria that unequivocally stratify risk among patients with *IDH*-mutant astrocytomas [2]. Although LGIMAs are less aggressive than grade 4 gliomas, their clinical outcomes vary widely and are difficult to predict; some tumors remain stable for extended periods, while others may progress to higher grades within months [8]. These observations suggest the need for more consistent classification criteria for LGIMAs, integrating both genetic and histopathological features for more accurate tumor characterization.

One gene that could be of particular relevance for LGIMA stratification is *TP53*. Among *TP53* mutations, R273C stands out in brain tumors, especially in *IDH*-mutant astrocytomas. This mutation is present in approximately 25% of *IDH*-mutant astrocytomas; it is significantly less prevalent in other cancer types, where, although also considered a hotspot mutation, it occurs in only 2.7% of cases [24,25,26].

In our initial cohort of 72 infiltrative astrocytic tumors, 14 were diagnosed as LGIMAs (ten were grade 2 and four were grade 3), all of which harbored *TP53* mutations. Of these, 33.3% carried the *TP53* R273C mutation. This proportion is slightly higher than that observed in the TCGA cohort (21.7%), although both values are consistent with previous reports of a 20–30% prevalence in *IDH*-mutant astrocytomas [3] and with the high frequency of this mutation in brain tissue (Figure 3b).

The R273C mutation is associated with worse prognosis, faster progression, and shorter survival, both in *IDH*-mutant astrocytomas and other cancers [3]. It is known that LGIMAs are characterized by low proliferative activity, typically reflected by low Ki-67 values. R273C is also linked to poor outcomes in these tumors, despite their relatively less aggressive nature [9]. In our LGIMA cohort, grade 3 tumors showed higher Ki-67 indices than grade 2 tumors. Interestingly, the tumors harboring the R273C mutation exhibited higher Ki-67 values than those of grade 2 and with other *TP53* mutations. In fact, tumors with the R273C mutation appear to constitute a category different from grade 2 and grade 3, according to the Ki67 profiles. This suggests that the current grade 2 classification may include tumors with higher biological aggressiveness that are not being accurately diagnosed. Our findings support the distinct role for R273C in grade 2 LGIMAs, making Ki-67 levels higher, possibly explaining the notably more aggressive behavior observed in these cases.

The higher frequency of *TP53* R273C in *IDH*-mutant astrocytomas compared to other *TP53* mutations could be related to the production of D-2-hydroxyglutarate (D-2HG) by these tumors. The neomorphic activity of the mutant IDH1/2 enzymes produces this metabolite, which promotes a hypermethylated state of DNA and histones [7,17], potentially favoring the occurrence of R273C. Enrichment of *TP53* R273C may occur via two possible mechanisms—selective mutagenesis or selective advantage—with the latter being, apparently, more likely [3].

Recent studies have shown that over 90% of all cancers with *TP53* mutations eventually lose the wild-type allele [19], consistent with *TP53*’s canonical role as a tumor suppressor. Marker et al. reported that all gliomas with the R273C mutation in their cohort, regardless of subtype or grade, lost their wild-type allele [3]. Our data, both from the CHGUV and TCGA cohorts, indicate that most R273C-mutant tumors were either homozygous for this mutation or harbored R273C in one allele and a different *TP53* mutation in the other (compound heterozygosis). A minority of cases exhibited R273C in only one allele, without other mutational findings. Two scenarios are possible here: (i) hemizygosity for *TP53* R273C with loss of the wild-type allele, consistent with tumor suppressor gene dynamics; (ii) true heterozygosity with retention of the wild-type allele. Based on our observations, the most probable scenario is loss of the wild-type allele in the vast majority of cases, resulting in only one allele carrying the R273C mutation.

Structural studies and in vitro models have demonstrated that the R273C mutation leads to a dramatic reduction in the DNA binding affinity of p53, with no p53-mediated transcriptional activity, although the protein retains wild-type stability [27,28]. This R273C p53 protein, having lost its original function, may gain novel functions with distinct biological effects in tumors. It is well established that loss of p53 activity is associated with increased tumor mutation burden [29]. In the case of R273C, this burden is higher than that for other *TP53* mutations in LGIMAs and has been linked to differential expression of up to 13 genes from the *HOX* family, which are involved in embryogenesis. The R273C p53 protein may contribute to these transcriptional changes [9]. Additionally, R273C has been implicated in mechanisms activating NF-κB, which may influence tumor behavior [30]. Nevertheless, the role of R273C remains incompletely understood, and its effects on various cancers are still under debate [12,14,15,31]. Mounting evidence suggests that p53 R273C may exert unique effects on tumor cell biology, conferring selective advantages to these cells within the tumor context [3].

Our data suggest a possible association between the *TP53* R273C mutation and female sex in patients with LGIMAs, as all five tumors with this mutation in our cohort occurred in female patients. Analysis of existing data from *IDH*-mutant astrocytomas of various grades in the TCGA database revealed a similar trend: R273C mutations were present in 26% of tumors in women compared to 11% in men [3]. In our LGIMA-specific cohort, the mutation was found in 55.5% of tumors from female patients (five of nine) and was absent in male tumors (zero of five), although the limited number of male cases may have been a confounding factor. To address this limitation, we examined the TCGA dataset. Among 115 *IDH*- and *TP53*-mutant LGIMAs, most tumors with the R273C mutation occurred in females, whereas other *TP53* mutations were more common in males. While these differences were not statistically significant in the TCGA dataset alone, combining our 14 LGIMAs with the TCGA cohort rendered the trends statistically significant. The increased frequency of *TP53* R273C in female LGIMA patients remains unexplained, although sex-based differences in gene expression or tumor microenvironments may play a role in selective pressures affecting LGIMAs.

As with glioblastomas and grade 4 astrocytomas, the classification of LGIMAs should be revised to incorporate genetic findings, alongside new refined and unambiguous histopathological criteria. Beyond *TP53* mutations, other recurrently mutated genes in LGIMAs include *ATRX*, *CIC*, *FUBP1*, *MUC16*, and *NOTCH*, as reported in the TCGA database. Additional relevant mutations may remain undiscovered, requiring more powerful genomic diagnostic tools. NGS is a modern approach that can aid in several ways: First, it can identify novel target genes missed by current testing strategies, but this requires broad gene panels that go beyond the most predictable glioma-associated genes. Second, it can distinguish between different mutations within the same gene, since, as demonstrated for R273C, specific mutations may have distinct biological implications. This may represent a major advancement over traditional histological techniques that detect only selected mutations. Third, NGS could improve the WHO classification, particularly for LGIMAs, and help explain why tumors of the same histological grade can behave so differently across patients.

In conclusion, we show that the *TP53* R273C mutation confers a distinct biological profile in LGIMAs, characterized by a level of cellular proliferation that might be intermediate between grades 2 and 3 (as measured by Ki-67) and more prevalent among female patients. These findings support a proposed update to the current WHO classification to incorporate the genetic profiles of LGIMAs, especially those harboring R273C, which may be underestimated and may require closer follow-up in order to provide more accurate diagnostic and prognostic guidance for patients.

## 4. Materials and Methods

### 4.1. Cohort Description

Fourteen low-grade, *IDH*-mutant astrocytomas were selected from the archives of the Department of Pathology, Consorcio Hospital General Universitario de Valencia (CHGUV), from a list of 72 adults with IDH-mutant astrocytomas. The cohort data were collected according to the protocol approved by the CHGUV Biobank (B.0001392), and this study was approved by the Institutional Research Ethics Committee of the CHGUV on 24 November 2023 (Registration No. 97/2023), based on the following selection criteria: adults over 18 years, grade 2 or 3 diffuse *IDH*-mutant astrocytomas, complete surgical resection, follow-up of more than 24 months, and available histological and molecular studies.

### 4.2. Clinical, Histological, and Immunohistochemical Data

Clinical and histological data were collected from pathology reports and information on clinical history: (i) clinical data: sex (male/female), patient age (years at diagnosis), clinical status (death due to disease, alive with disease, alive free of disease, relapses), and survival in months; (ii) histological data: resected tumor size, histological grade, mitotic rate in 2 mm^2^, % Ki-67 (proliferative index), % p53 neoplastic nuclei, ATRX neoplastic nuclear expression, and IDH1 neoplastic nuclear expression; (iii) FISH: 1p/19q codeletion. Slides were reviewed by two pathologists. Briefly, tumors were formalin-fixed and paraffin-embedded, and sections were stained with hematoxylin and eosin. An immunohistochemical study was carried using a Ventana Autostainer (Roche Diagnostics, Barcelona, Spain) with Ventana antibodies (IDH1 antibody (Ref: MAD-00047SGLD; clone H09; 1.5 μg/mL); p53 antibody (Ref: (92)800-2912; clone DO-7; 0.5 μg/mL); anti-Ki-67 antibody (Ref: (92)790-4286, clone 30-9, 2 μg/mL)) and a Sigma-Aldrich (Sigma-Aldrich, St. Louis, MO, USA) antibody (ATRX (Ref: HPA001906-100; rabbit polyclonal; 1/50 dilution)). The FISH study on 1p/19q was conducted using paraffin sections from 7 cases, using LSI 1p36/19q13 probe sets from Vysis (Abbott Laboratories, Downers Grove, IL, USA).

### 4.3. Molecular Studies

Molecular analyses were performed using the Oncomine^TM^ Precision Assay next-generation sequencing panel (Thermo Fisher Scientific, Valencia, Spain). This panel detects hotspot mutations (substitutions, insertions, and deletions), copy number variations (CNVs), and gene fusions across 50 cancer driver genes.

In order to compare our CHGUV cohort with other larger cohorts, we studied additional LGIMA cases from the Cancer Genome Atlas (TCGA, https://www.cancer.gov/ccg/research/genome-sequencing/tcga, accessed on 14 July 2025). We selected a cohort with 115 LGIMA cases with *IDH1/2* and *TP53* mutated (TCGA cohort). Molecular data were obtained from cBioPortal (https://www.cbioportal.org, last accessed 29 April 2025).

### 4.4. Statistical Analysis

Statistical analyses were performed using GraphPad Prism software, version 8.0.1. (San Diego, CA, USA), with the results presented as the mean ± S.D. These analyses were conducted using one-way analysis of variance (ANOVA) followed by Student’s *t*-test for dual comparisons. The chi-squared test was used to examine the correlation between *TP53* mutations and the sex of the patients. A *p*-value < 0.05 was considered statistically significant.

## Figures and Tables

**Table 1:** Tumor grades, sex, age, relevant mutations, follow-up after surgery, and comorbidities of the 14 patients and LGIMAs.

Case	Grade	Sex	Age	*IDH1/2* Mut.	*TP53* Mut.	Follow-Up After Surgery (Months)	Comorbidities
1	3	W	59	*IDH1* R132H	R273C	36	-
2	2	W	52	*IDH2* R172G	R273C	45	-
3	2	W	29	*IDH1* R132G	R273C	54	-
4	2	W	66	*IDH1* R132H	R273C	33	-
5	2	W	52	*IDH1* R132H	R273C	49	Breast cancer
6	2	M	35	*IDH1* R132H	R248Q	168	-
7	3	M	47	*IDH1* R132H	R248Q	168	-
8	2	M	70	*IDH1* R132H	R248Q	24	-
9	3	W	45	*IDH1* R132H	R175H	39	-
10	3	W	39	*IDH1* R132H	R175H	25	-
11	2	M	39	*IDH1* R132H	R273H	240	Cutaneous T-cell lymph.
12	2	M	34	*IDH1* R132H	R273L	41	-
13	2	W	30	*IDH1* R132H	G245D	204	-
14	2	W	42	*IDH1* R132L	H179R, I195T	108	-

**Table 2:** Comparison of clinical data between LGIMAs with *TP53* R273C mutation and LGIMAs with other *TP53* mutations, from our cohort.

		LGIMAs (Grades 2 and 3)	
	R273C	Other *TP53* Mut	All
Sex (men/women)	0/5	5/4	5/9
Grade (2/3)	4/1	6/3	10/4
Age (µ ± SD)	51.6 ± 13.9	42.3 ± 5.4	45.6 ± 12.8
Tumor size (cm^3^) (µ ± SD)	34.4 ± 51.3	32.7 ± 55.7	33.3 ± 52.2
Ki-67	5.6 ± 3.2	5.2 ± 3.6	5.4 ± 3.4

## Data Availability

The raw data supporting the conclusions of this article will be made available by the authors on request.

## Abbreviations

The following abbreviations are used in this manuscript:

CHGUV	Consorcio Hospital General Universitario de Valencia
CNS	Central nervous system
IDH	Isocitrate dehydrogenase
IMA	IDH-mutant astrocytoma
LGIMA	Low-grade, IDH-mutant astrocytoma
NGS	Next-generation sequencing
TCGA	The Cancer Genome Atlas
WHO	World Health Organization

## References

[1] Rajesh Y., Pal I., Banik P., Chakraborty S., Borkar S.A., Dey G., Mukherjee A., Mandal M. (2017). Insights into Molecular Therapy of Glioma: Current Challenges and next Generation Blueprint. Acta Pharmacol. Sin..

[2] Louis D.N., Perry A., Wesseling P., Brat D.J., Cree I.A., Figarella-Branger D., Hawkins C., Ng H.K., Pfister S.M., Reifenberger G. (2021). The 2021 WHO Classification of Tumors of the Central Nervous System: A Summary. Neuro Oncol..

[3] Marker D.F., Agnihotri S., Amankulor N., Murdoch G.H., Pearce T.M. (2022). The Dominant TP53 Hotspot Mutation in IDH -Mutant Astrocytoma, R273C, Has Distinctive Pathologic Features and Sex-Specific Prognostic Implications. Neuro Oncol. Adv..

[4] Suzuki H., Aoki K., Chiba K., Sato Y., Shiozawa Y., Shiraishi Y., Shimamura T., Niida A., Motomura K., Ohka F. (2015). Mutational Landscape and Clonal Architecture in Grade II and III Gliomas. Nat. Genet..

[5] Gao J., Aksoy B.A., Dogrusoz U., Dresdner G., Gross B., Sumer S.O., Sun Y., Jacobsen A., Sinha R., Larsson E. (2013). Integrative Analysis of Complex Cancer Genomics and Clinical Profiles Using the cBioPortal. Sci. Signal..

[6] Berzero G., Di Stefano A.L., Ronchi S., Bielle F., Villa C., Guillerm E., Capelle L., Mathon B., Laurenge A., Giry M. (2021). IDH-Wildtype Lower-Grade Diffuse Gliomas: The Importance of Histological Grade and Molecular Assessment for Prognostic Stratification. Neuro Oncol..

[7] Cairns R.A., Mak T.W. (2013). Oncogenic Isocitrate Dehydrogenase Mutations: Mechanisms, Models, and Clinical Opportunities. Cancer Discov..

[8] van den Bent M.J., Brandes A.A., Taphoorn M.J.B., Kros J.M., Kouwenhoven M.C.M., Delattre J.-Y., Bernsen H.J.J.A., Frenay M., Tijssen C.C., Grisold W. (2013). Adjuvant Procarbazine, Lomustine, and Vincristine Chemotherapy in Newly Diagnosed Anaplastic Oligodendroglioma: Long-Term Follow-up of EORTC Brain Tumor Group Study 26951. J. Clin. Oncol..

[9] Zhang J., Liu M., Fang Y., Li J., Chen Y., Jiao S. (2022). TP53 R273C Mutation Is Associated With Poor Prognosis in LGG Patients. Front. Genet..

[10] Petitjean A., Mathe E., Kato S., Ishioka C., Tavtigian S.V., Hainaut P., Olivier M. (2007). Impact of Mutant P53 Functional Properties on TP53 Mutation Patterns and Tumor Phenotype: Lessons from Recent Developments in the IARC TP53 Database. Hum. Mutat..

[11] Salnikova L.E. (2014). Clinicopathologic Characteristics of Brain Tumors Are Associated with the Presence and Patterns of TP53 Mutations: Evidence from the IARC TP53 Database. NeuroMolecular Med..

[12] Chan K.-T., Lung M.L. (2004). Mutant P53 Expression Enhances Drug Resistance in a Hepatocellular Carcinoma Cell Line. Cancer Chemother. Pharmacol..

[13] Zhang C., Li Z., Qi F., Hu X., Luo J. (2019). Exploration of the Relationships between Tumor Mutation Burden with Immune Infiltrates in Clear Cell Renal Cell Carcinoma. Ann. Transl. Med..

[14] Rasti M., Azimi T. (2015). TP53 Binding to BRCA1 and RAD51 in MCF7 and MDA-MB-468 Breast Cancer Cell Lines In Vivo and In Vitro. Avicenna J. Med. Biotechnol..

[15] Román-Rosales A.A., García-Villa E., Herrera L.A., Gariglio P., Díaz-Chávez J. (2018). Mutant P53 Gain of Function Induces HER2 Over-Expression in Cancer Cells. BMC Cancer.

[16] Chen Y.-J., Hakin-Smith V., Teo M., Xinarianos G.E., Jellinek D.A., Carroll T., McDowell D., MacFarlane M.R., Boet R., Baguley B.C. (2006). Association of Mutant TP53 with Alternative Lengthening of Telomeres and Favorable Prognosis in Glioma. Cancer Res..

[17] Han S., Liu Y., Cai S.J., Qian M., Ding J., Larion M., Gilbert M.R., Yang C. (2020). IDH Mutation in Glioma: Molecular Mechanisms and Potential Therapeutic Targets. Br. J. Cancer.

[18] Singh A., Gurav M., Dhanavade S., Shetty O., Epari S. (2017). Diffuse Glioma—Rare Homozygous IDH Point Mutation, Is It an Oncogenetic Mechanism?. Neuropathology.

[19] Donehower L.A., Soussi T., Korkut A., Liu Y., Schultz A., Cardenas M., Li X., Babur O., Hsu T.-K., Lichtarge O. (2019). Integrated Analysis of TP53 Gene and Pathway Alterations in The Cancer Genome Atlas. Cell Rep..

[20] Wesseling P., Capper D. (2018). WHO 2016 Classification of Gliomas. Neuropathol. Appl. Neurobiol..

[21] Galbraith K., Snuderl M. (2021). Molecular Pathology of Gliomas. Surg. Pathol. Clin..

[22] Zhang Y., Dube C., Gibert M., Cruickshanks N., Wang B., Coughlan M., Yang Y., Setiady I., Deveau C., Saoud K. (2018). The P53 Pathway in Glioblastoma. Cancers.

[23] López-Ginés C., Muñoz-Hidalgo L., San-Miguel T., Megías J., Triviño J.C., Calabuig S., Roldán P., Cerdá-Nicolás M., Monleón D. (2021). Whole-Exome Sequencing, EGFR Amplification and Infiltration Patterns in Human Glioblastoma. Am. J. Cancer Res..

[24] Li J., Yang L., Gaur S., Zhang K., Wu X., Yuan Y.-C., Li H., Hu S., Weng Y., Yen Y. (2014). Mutants TP53 p.R273H and p.R273C but Not p.R273G Enhance Cancer Cell Malignancy. Hum. Mutat..

[25] Baugh E.H., Ke H., Levine A.J., Bonneau R.A., Chan C.S. (2018). Why Are There Hotspot Mutations in the TP53 Gene in Human Cancers?. Cell Death Differ..

[26] Freed-Pastor W.A., Prives C. (2012). Mutant P53: One Name, Many Proteins. Genes Dev..

[27] Eldar A., Rozenberg H., Diskin-Posner Y., Rohs R., Shakked Z. (2013). Structural Studies of P53 Inactivation by DNA-Contact Mutations and Its Rescue by Suppressor Mutations via Alternative Protein-DNA Interactions. Nucleic Acids Res..

[28] Baroni T.E., Wang T., Qian H., Dearth L.R., Truong L.N., Zeng J., Denes A.E., Chen S.W., Brachmann R.K. (2004). A Global Suppressor Motif for P53 Cancer Mutants. Proc. Natl. Acad. Sci. USA.

[29] Chalmers Z.R., Connelly C.F., Fabrizio D., Gay L., Ali S.M., Ennis R., Schrock A., Campbell B., Shlien A., Chmielecki J. (2017). Analysis of 100,000 Human Cancer Genomes Reveals the Landscape of Tumor Mutational Burden. Genome Med..

[30] Soubannier V., Stifani S. (2017). NF-κB Signalling in Glioblastoma. Biomedicines.

[31] Zhang D., Zhang W., Liu W., Mao Y., Fu Z., Liu J., Huang W., Zhang Z., An D., Li B. (2017). Human Papillomavirus Infection Increases the Chemoradiation Response of Esophageal Squamous Cell Carcinoma Based on *P53* Mutation. Radiother. Oncol..

